# Polyhydroxyalkanoate accumulation in Streptomyces coelicolor affected by SCO7613 gene region

**DOI:** 10.3906/biy-2011-16

**Published:** 2021-06-23

**Authors:** Zeynep DEMİR ÖKSÜZ, Tuğrul DORUK, Nevin YAĞCI, Sedef TUNCA GEDİK

**Affiliations:** 1 Molecular Biology and Genetic Department, Faculty of Science, Gebze Technical University, Kocaeli Turkey; 2 Department of Environmental Engineering, Faculty of Civil Engineering, İstanbul Technical University, İstanbul Turkey; 3 Molecular Biology and Genetic Department, Faculty of Arts and Science, Ondokuz Mayıs University, Samsun Turkey

**Keywords:** *Streptomyces coelicolor*, SCO7613, polyhydroxyalkanoate synthase, *pha* C, glycerol

## Abstract

Polyhydroxyalkanoate (PHA) is stored as an important carbon and energy source in bacterial cells. For biomedical applications, gram-positive bacteria can be better sources of PHAs, since they lack outer membrane lipopolysaccharide. Although gram-positive *Streptomyces coelicolor* A3(2) has been indicated as a high potential PHA producer, *pha* C gene that encodes the key enzyme PHA synthase in the metabolic pathway is not determined in its genome. BLAST search results of the GenBank database argued that SCO7613 could specify a putative polyhydroxyalkanoate synthase (PhaC) responsible for PHA biosynthesis. Deduced amino acid sequence of SCO7613 showed the presence of conserved lipase box like sequence, ^555^GASAG^559^, in which serine residue was present as the active nucleophile. Present study describes deletion of putative *S. coelicolor pha* C gene via PCR dependent method. We showed that SCO7613 is not an essential gene in *S. coelicolor* and its deletion affected PHA accumulation negatively although it is not ceased. Transcomplementation abolished the mutant phenotype, demonstrating that the decrease in PHA resulted from the deletion of SCO7613.

## 1. Introduction

Polyhydroxyalkanoates (PHAs) are biodegradable and biocompatible polymers of 3-, 4-, 5- and 6 hydroxyalkanoic acids (HA). They are gaining more commercial importance due to their potential as substitutes for synthetic plastics (Anderson and Dawes, 1990). Moreover, the studies conducted in the last decade draw attention to the potential usage of PHAs in the medical field because of their biocompatibility, mechanical stability, and strength (De Souza and Shivakumar, 2019). Microorganisms are the main source of these polymers since their chemical synthesis is not feasible. When there is excess carbon under nutrient deprivation conditions many bacteria synthesize PHAs (Reddy et al., 2003). PHAs are produced on a large scale by gram-negative bacteria. However, gram-positive bacteria are better PHA sources for the medical field, since they lack outer membrane lipopolysaccharide (LPS), which induces strong immunogenic reactions (Lee, 1996). 

As gram-positive bacteria, *Streptomyces* spp., which are capable of synthesizing a variety of primary and secondary metabolites, are known to accumulate also PHAs (Kannan and Rehacek, 1970; Manna et al., 1999; Verma et al., 2002). Krishnan et al. (2017) have shown that this group of bacteria could be potential source for theproduction of PHA with desirable characteristics. Especially *Streptomyces coelicolor* A3(2) was shown to be a candidate organism that can be transformed into a novel PHA producer by genetic engineering (Kalia et al., 2007).

**Figure 1:**
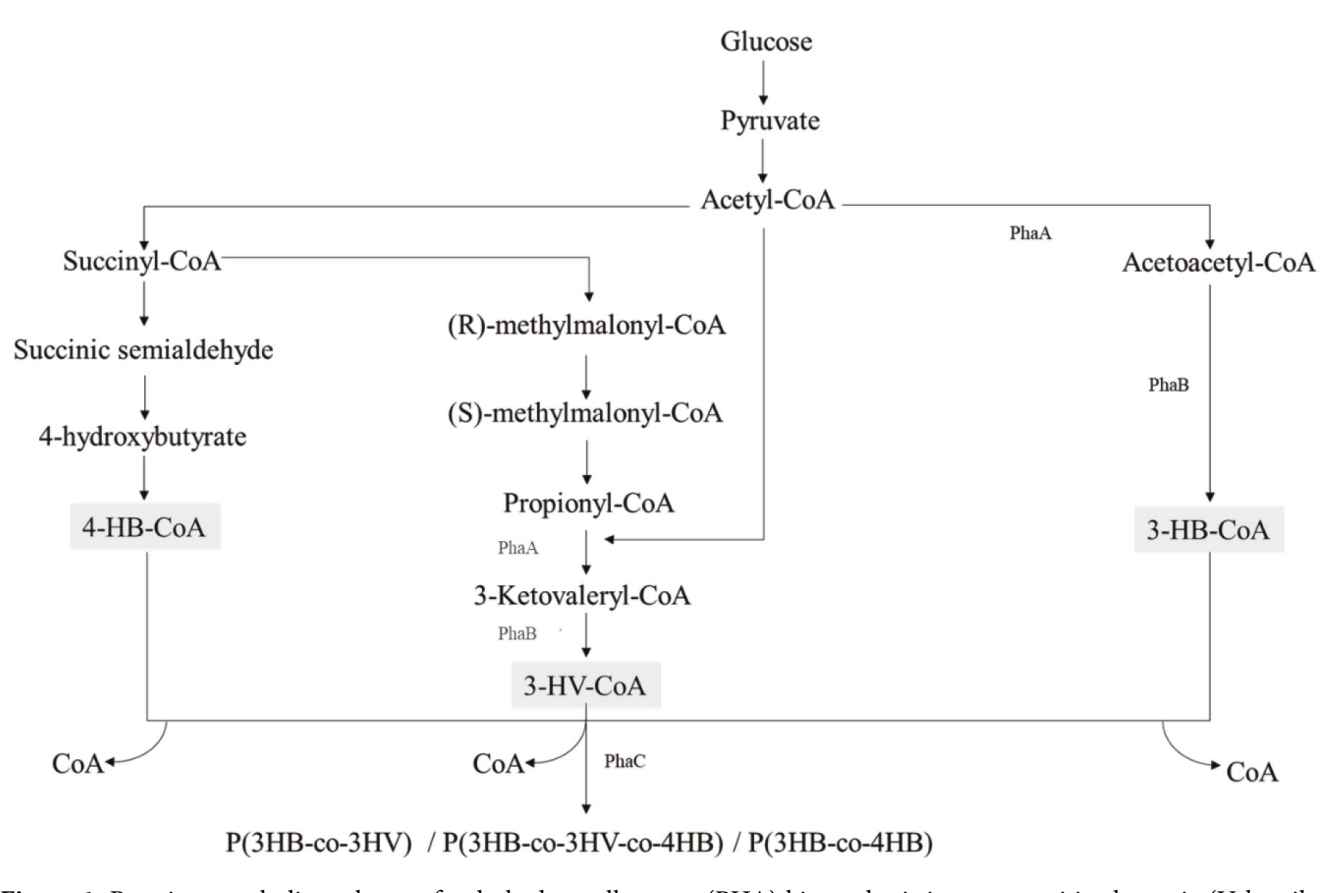
Putative metabolic pathway of poly-hydroxyalkanoate (PHA) biosynthesis in gram positive bacteria (Valappil et al., 2007). The last enzyme of the pathway, PHA synthase (PhaC), is responsible for the production of 3HB, 3HV and 4HB.

A putative metabolic pathway for the synthesis of PHAs by gram-positive bacteria was shown by Valappil et al. (2007). In short, acetyl-CoA, which is produced by the degradationof glucose, can be converted to succinyl CoA and after few reactions 4-hydroxybutyryl-CoA (4-HB-CoA) is produced. In another pathway acetyl-CoA is first converted to acetoacetyl CoA and 3-hydroxybutryl-CoA (3-HB-CoA) is formed. Acetyl-CoA also condenses with propionyl-CoA giving rise to first 3-ketovaleryl-CoA and later 3-hydroxyvaleryl-CoA (3-HV-CoA) is produced (Figure 1). Finally, PHA synthase polymerizes these CoA thioesters of HA into polyhydroxyalkanoates (Figure 1). Recently, engineered PHA synthases are in use to synthesize new biopolymers (Zou et al., 2017).

Although the genes responsible for PHA synthesis are usually clustered in the genomes of bacteria ( *Ralstonia eutropha, Alcaligenes latus, Chromobacterium violaceum* ), there are exceptions in which *pha* C is not a part of a cluster with other *pha* genes ( *Nocardia corallina, Rhodococcus ruber, Rickettsia prowazekii* and *Aeromonas caviae* ) (Rehm, 2003). Knowledge about the genes responsible for the biosythesis of PHA in *Streptomyces* is limited. Although there are some genes assigned for a product [(methylmalonyl-CoA mutase (NP_630903), putative methylmalonylCoA epimerase (NP_624722/NP_627536/NP_627930) and putative methylmalonyl-CoA decarboxylase (NP_629669), succinic semialdehyde dehydrogenase (CAD55522)], there are still some of them waiting to be identified. *S. coelicolor* A3(2) was pointed out to have a high potential to be transformed into an important PHA producer (Kalia et al., 2007), however even its *pha* C gene that encodes the key enzyme PHA synthase is not identified in its genome. The same is true for acetyl-CoA acetyltransferase encoding *pha* A gene and acetoacetyl-CoA reductase encoding *pha* B gene (Figure 1). 

In the present study, we aimed to identify *pha* C gene of *S. coelicolor* A3(2). Our bioinformatic analysis has shown that no gene region perfectly matches with the defined *pha* C genes from other microorganisms. To the best of our knowledge, SCO7613 gene region (Genbank accession number: AL645882.2) of *S. coelicolor* A3(2) was the only DNA sequence that shows 70% and 75% similarity with the 5’ and 3’ ends of *Streptomyces aureofaciens pha* C gene (Accession Number: AY032926.1), respectively. We deleted SCO7613 by using a PCR dependent method and trans-complemented the mutation in this study.

## 2. Materials and methods

### 2.1. Plasmids, bacteria, media and culture conditions

Bacteria and plasmids used in this work are represented in Table 1. TSB, YEME, nitrogen basal medium and R2YE media were used to grow *S. coelicolor* strains. LB liquid and solid media were used to culture *E. coli* strains. The growth temperature for *Streptomyces* strains was 30 °C. *E. coli* strains were cultivated at 30 or 37 °C. pIJ790 plasmid, which is responsible for recombination, and St2H2 cosmid were propagated in *E. coli* BW25113. As the nonmethylating plasmid donor *E. coli* ET12567 was used in the conjugation experiments. Antibiotics [ampicillin (100 µg/mL), chloramphenicol (25 µg/mL), apramycin (50 µg/mL) and kanamycin (50 µg/mL)] were used when necessary.

**Table 1:** Bacteria and cosmid/plasmid.

Bacterial strains/plasmids/cosmids	Genotype	Reference
pHZ1351	tsr Ltz− sti+	Kieser et al. (2000)
St2H2	Cosmid containing SCO7613 gene region	Sanger Institute
pIJ773	aac(3)IV	Gust et al. (2003)
pUZ8002	tra, neo, RP4	Paget et al. (1999)
pIJ790	l-RED (gam, bet, exo), cat, araC, rep101ts	Gust et al. (2003)
pHZ-7613	pHZ1351 containing SCO7613 gene region	This study
E. coli strains		
E. coli BW25113	K12 derivative: ΔaraBAD, ΔrhaBAD	Datsenko and Wanner (2000)
E. coli ET12567	dam, dcm, hsdS, cat, tet	MacNeil et al. (1992)
Streptomyces strains		
S. coelicolor A3(2)	Wild-type prototrophic	Hopwood (1999)
S. coelicolor ΔSCO7613	Strain without SCO7613 gene region	This study
S. coelicolor ΔSCO7613+pHZ-7613	ΔSCO7613 transformed with pHZ-7613	This study

### 2.2. DNA methods

Cosmid and plasmid isolations, restriction enzyme digestions and Southern blot analysis were performed according to Sambrook et al. (1989). Probe DNAs of SCO7613 and apramycin resistance ( *aac* (3)IV) genes were obtained by PCR by using specific primers (Table 2) and they were labelled with digoxigenin using DIG DNA labelling mix (Roche Biochemicals). *Streptomyces* total DNA isolation, transformation of *Streptomyces* and *E. coli,* intergeneric conjugation were as described by Kieser et al., (2000). 

**Table 2:** Primers used in this study.

Name	Sequence (5’to 3’)
Apr-1F	CATGGCGGCCCCGCGACCTGATCCGCCGGACAGCGCCTATGTAGGCTGGAGCTGCTTC
Apr-1R	ATGTCCCGACGCGTGGCGAGGACCACACTCCGAGGCATGATTCCGGGGATCCGTCGACC
Apr-2F	ATTCCGGGGATCCGTCGACC
Apr-2R	TGTAGGCTGGAGCTGCTTC
SCO7613-1F	ACCCGGGCCGAGAACCAGTG
SCO7613-1R	GCGGCCGCCCTTGTCCT

### 2.3. Deletion of SCO7613 

Apramycin resistance gene ( *aac* (3)IV) was used to replace *S. coelicolor* SCO7613 by PCR based method (Gust et al., 2002). Long primers (Table 2) were used to amplify “gene disruption cassette” containing both *aac* (3)IV gene and oriT region by using pIJ773 plasmid as a template. *E. coli* BW25113⁄pIJ790 were first transformed with St2H2 cosmid and then the disruption cassette was used to transform these cells. Mutation in the cosmid was proven by both restriction enzyme digestion and polymerase chain reaction. Then the mutant cosmid was transferred to *S. coelicolor* by conjugation with *E. coli* ET12567/pUZ8002. Apr^R^ and Kn^S^ conjugants were selected. 

### 2.4. Complementation of ΔSCO7613 strain

A 3700 bp *Bam* HI- *Sac* I fragment of St2H2 cosmid that contains SCO7613 gene region was extracted from agarose gel and ligated with pHZ1351 vector, predigested with *Bam* HI- *Sac* I. The recombinant plasmid (pHZ-7613) was obtained in *E. coli* DH5α. Recombinant plasmid isolated from methylation deficient strain *E. coli* 12567 used to transform protoplasts of *S. coelicolor* ΔSCO7613 strain (Kieser et al., 2000). 

### 2.5. Purification of PHA for spectrophotometric and FTIR analysis

Purification of PHA was performed according to Law and Slepecky (1961) with small modifications. *Streptomyces* cells were grown in 100 mL of Nitrogen basal medium for 72 h. After centrifugation at 2000 g for 20 min, pellet was collected and washed with distilled water and dried at 80 °C for 4 days. Then the pellet was resuspended in 50 mL sodium hypochlorite solution and incubated for 12 h at 37 °C. Pellets were collected by centrifugation **(** 2000 g, 30 min) and they were first washed with water, then with acetone, ethanol and diethyl ether in order. Finally, the polymer was extracted by boiling chloroform for 10 min. After filtration with Whatman No.1 filter (Cat No: 1001-010), chloroform was evaporated to dryness at room temperature. The polymer was used for spectrophotometric and FTIR analysis.

### 2.6. Purification of PHA for GC analysis

*Streptomyces* cells, grown in 100 mL Nitrogen basal medium (Kieser et al., 2000) for 72 h, were centrifuged at 2000 g for 20 min. After centrifugation at 2000 g for 20 min, the pellet was resuspended in 10 mL distilled water and lyophilized at **–** 40 **°** C for 5 h. The temperature was increased to 20 °C and the samples were dried for 24 h under 10-pascal pressure.

### 2.7. Measurement of PHA

#### 2.7.1. Spectrophotometric analysis 

Purified polymer which is prepared from the samples with same wet weight was dissolved in 10 mL H_2_SO_4_ and heated for 10 min in boiling water to convert PHB to crotonic acid which was then measured spectrophotometrically (Schimadzu CPS-240a) at 235 nm (Manna et al., 1999). A standard curve was prepared with pure PHB and PHB concentration of the sampleswas determined by using this curve (Law and Slepecky, 1961). 

#### 2.7.2. FTIR (Fourier transform infrared spectroscopy) analysis

The purified solid PHA from *S. coelicolor* strains obtained as described in subsection 2.5 were used directly in the FTIR analysis. Perkin Elmer (USA) model 100 FTIR was used for spectroscopic analysis where the scan range was 4000 **–** 650 cm **^–^** ^1^ (scan number: 4; scan speed: 0.5 cm s **^–^** ^1^).

#### 2.7.3. GC analysis

A gas chromatograph (Agilent 6890 N) equipped with a 0.53 mm diameter capillary column (J&W DB-FFAP FID) with 1 μm film thickness and 30 m length was used for GC analysis. Auto-sampler and a flame ionization detector were also part of the chromatograph. The temperature of the injector and the detector was 180 **°** C and 250 °C, respectively. Temperature program of the column was: initial temperature was set to 60 °C for 4 min, then increased to 220 °C in 12 min and holded at this temperature for 6 min. Caproic acid sodium salt was the standard for 3H2MV and 3- hydroxybutyricacid-co-3-hydroxyvaleric acid was the standard for PHB and PHV. The total polyhydroxyalkanoate present in the sludge was calculated as a percentage of VSS (volatile suspended solids) on cell dry weight (CDW). According to the oxidation stoichiometry PHA concentration (mg/L) was converted to mg COD/L: 1.67 mgCOD/mgPHB for PHB and 1.92 mgCOD/mgPHV for PHV where COD is chemical oxygen demand (Beccari et al., 2009).

## 3. Results

### 3.1. Analysis of the S. coelicolor SCO7613 gene region

**Figure 2:**
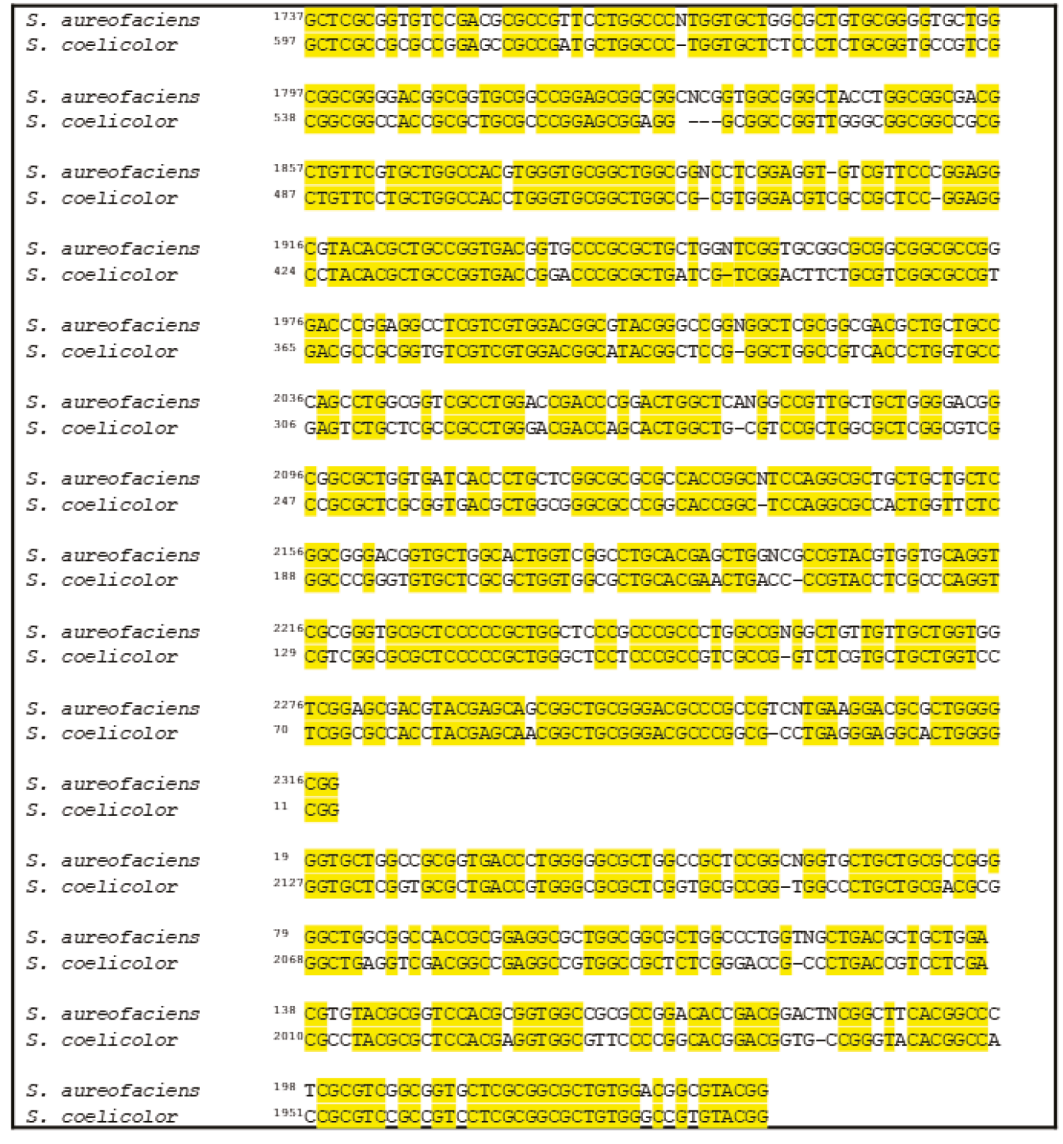
Alignment of PHA synthase gene of S. aureofaciens with SCO7613 of S. coelicolor. The consensus sequences are shown in yellow color.

**Figure 3:**
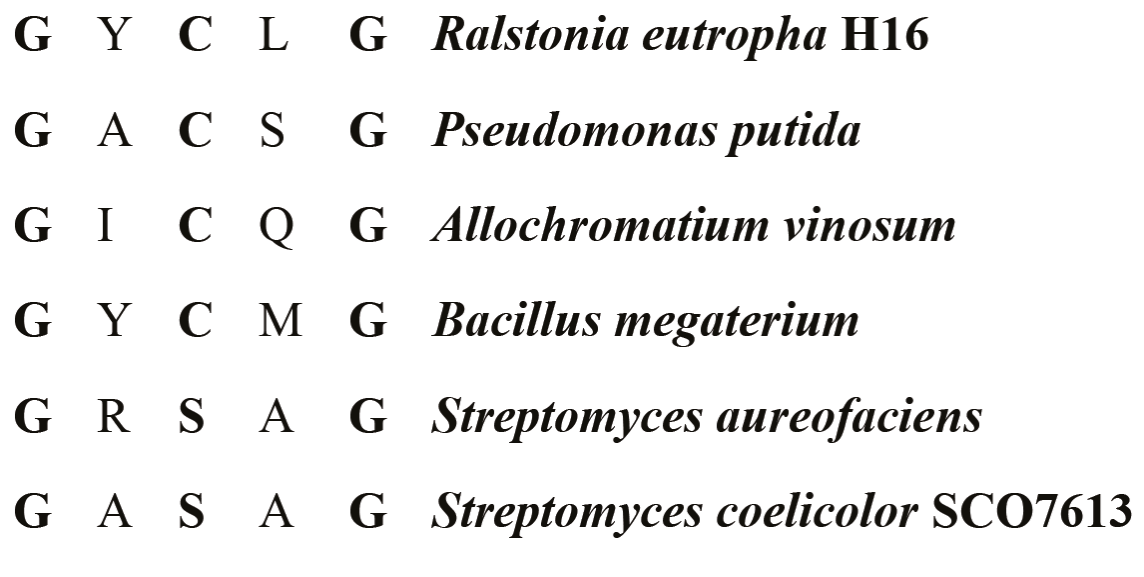
Comparison of putative lipase box-like sequence of SCO7613 with the lipase box-like sequences of other bacteria (Ilham et al., 2014; Ramachander and Rawal, 2005; Rehm, 2003).

BLAST searches of the GeneBank database argued that SCO7613 of *S. coelicolor* , shows 70% and 75% similarity with the 5’end and 3’ end of *S. aureofaciens* ’s *pha* C gene, respectively (Figure 2). The deduced translation product of SCO7613 encodes for a protein, which shows 44% similarity to the *S. aureofaciens* PhaC, with a calculated molecular mass of 81.9 kDa. In the active site of PhaC synthases usually a lipase box-like sequence (Gly-X-Cys-X-Gly) is present (Figure 3). A putative lipase box in SCO7613 is determined to be ^555^GASAG^560^. Similar to *S. aureofaciens* PhaC, serine residue is present instead of cystein as the active nucleophile in the possible lipase box. Examination of upstream and downstream regions of the *S. coelicolor* SCO7613 showed that putative regulatory protein (SCO7614) is located in front of SCO7613, while conserved hypothetical protein is behind (SCO7612).

### 3.2. Deletion of SCO7613 from S. coelicolor genome

**Figure 4:**
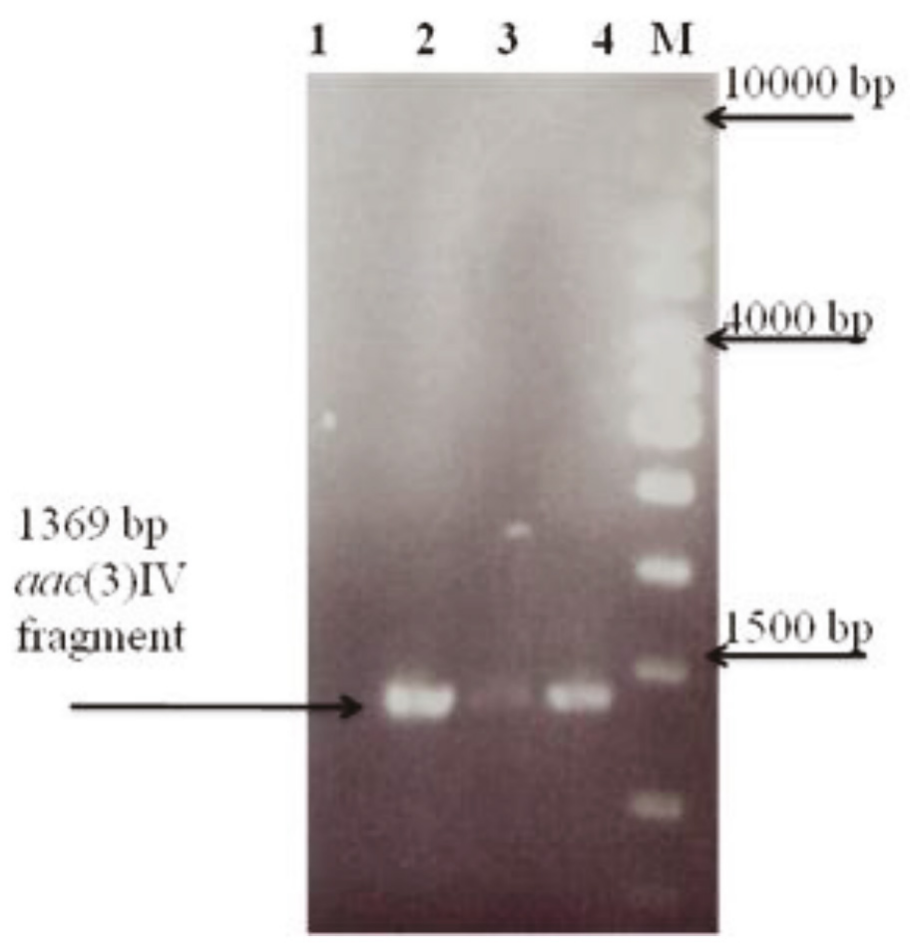
Verification of mutation by PCR by using aac(3) IV primers. 1: wild type; 2,3: putative double cross over mutant (ΔSCO7613); 4: single cross over mutant; M: 1 kb DNA marker (Bioron).

**Figure 5:**
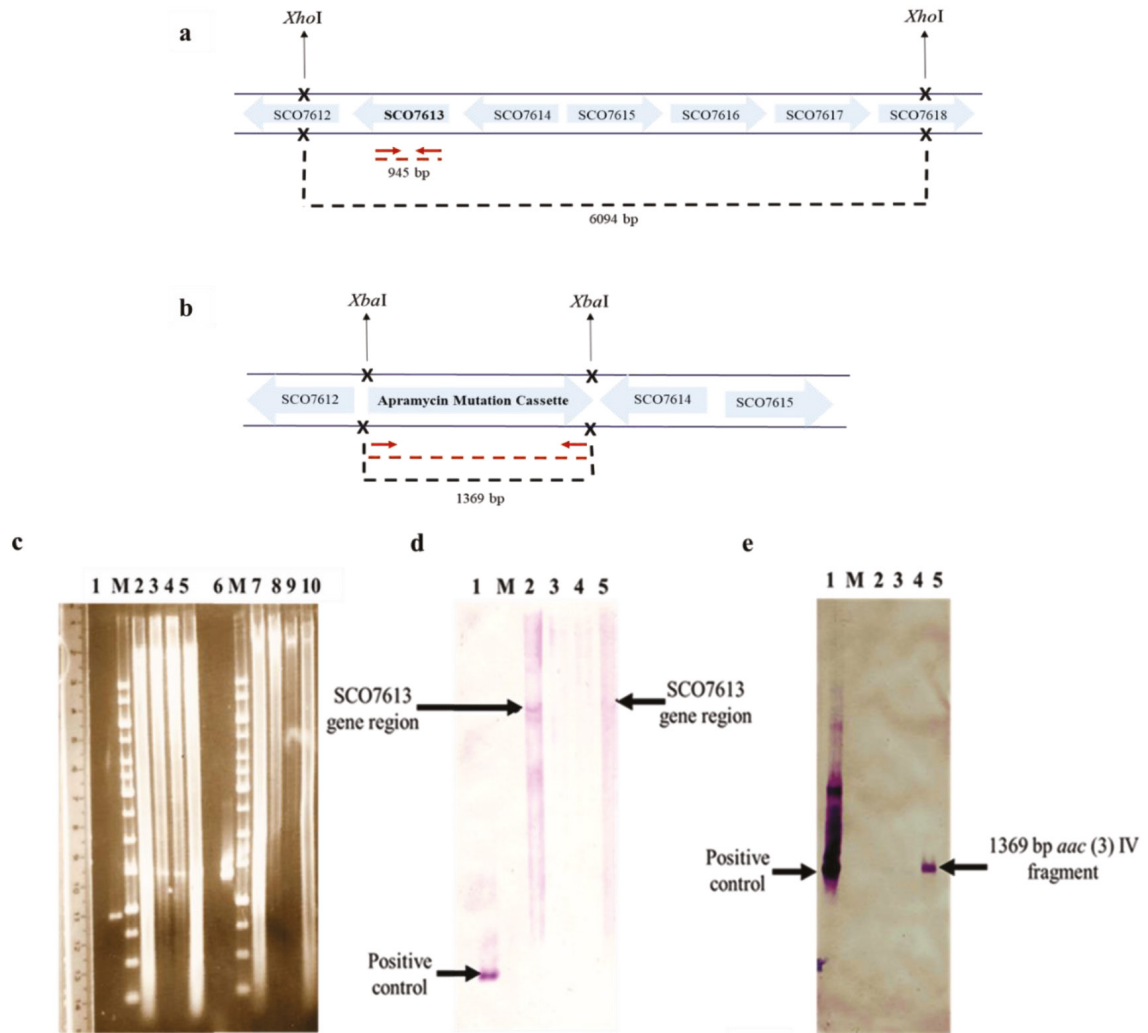
Southern blot hybridization that shows deletion of SCO7613. Hybridization sites of the SCO7613 probe in the S. coelicolor A3(2) genome(a) and apramycin probe in the genome of ΔSCO7613 (b) are illustrated with red arrows. Black crosses indicate XhoI and XbaI restriction enzyme cutting sites. Red-Safe stained agarose gel showing; 945 bp SCO7613 (lane 1) and 1369 bpaac(3) IV gene fragments (lane 6) as positive controls and XhoI-digested chromosomal DNA of the wild-type (lane 2), the double cross over mutants (lanes 3-4), the single cross over mutant (lane 5) and XbaI-digested chromosomal DNA of the wild-type (lane 7), the double cross over mutants (lanes 8–9), the single cross over mutant (lane10) (c). Hybridization results of XhoI-digested wild-type’s genome (lane 2), the double cross over mutants’ genome (lanes 3–4), the single cross over mutant’s genome (lane 5) probed with the 945 bp SCO7613 fragment (d). Hybridization pattern of XbaI-digested chromosomal DNA of the wild-type S. coelicolor (lane 2), the double cross over mutants (lanes 3–4), the single cross over mutant (lane 5) probed with the 1369 bpaac(3) IV gene fragment (e). 1 kb DNA marker (Bioron).

SCO7613 gene region of *S. coelicolor* was deleted by using a PCR based method in which a cassette containing apramycin gene was replaced the target gene. Two Apr^R^-Kn^S^ conjugants (double cross over mutants) and one Apr^R^-Kn^R^ conjugant (single cross over mutant) were selected and mutation was confirmed by both PCR amplification (Figure 4) and Southern blot hybridization (Figure 5). A 1369 bp DNA fragment was obtained only in the mutant strain when *aac* (3)IV (apramycin resistance gene) specific primers were used in PCR reactions (Figure 4). Figures 5a and 5b show hybridization sites of both SCO7613 and *aac* (3)IV probes in the genome of *S. coelicolor* . The DNA samples used in the Southern blot experiment were shown in Figure 5c. A 6094 bp hybridization band was obtained only for the wild-type and single cross over mutant strains when SCO7613 gene fragment was used as the probe (Figure 5d). Hybridization with the probe specific for apramycin resistant gene resulted in a band of 1369 bp for the ∆SCO7613 and single cross over mutant strains (Figure 5e). For complementation of mutation, pHZ-7613 recombinant plasmid has been prepared in *E. coli* by ligating a 3700 bp *Bam* HI- *Sac* I fragment of St2H2 cosmid with pHZ1351 digested with the same enzymes. Then the recombinant plasmid was transferred into ΔSCO7613 strain.

### 3.3. PHA production by S. coelicolor ΔSCO7613

Deletion of the SCO7613 did not affect the growth of the mutant strain negatively, even mutant strain was growing better than the wild type (Figure 6). Specific PHA production by the wild type, ΔSCO7613, and the mutant complemented with pHZ-7613 was compared by using spectrophotometric (Figure 7), gas chromatography (GC) (Table 3) and FTIR (Fourier transform infrared spectroscopy) analysis (Figure 8). PHA content of the ΔSCO7613 strain was found to be 41.2% less than the wild type by using spectrophotometric measurement and this decrease has been trans-complemented by pHZ-7613 (Figure 7). GC results also confirmed that PHA content of the ΔSCO7613 strain was less than the wild type and complementation of ΔSCO7613 mutation with pHZ-7613 plasmid increased PHA production in this strain (Table 3). These results proved that the deletion of SCO7613 caused a considerable decrease in PHA production in the mutant strain. Different sets of experiments gave the same results. 

**Table 3:** Determination of the PHA production by S. coelicolor (WT), ΔSCO7613 and ΔSCO7613+pHZ-7613 strains by using Gas chromatography.PHA(mg/L)PHA (mg COD/L)PHA (mmol C/L)PHA (mg C/L)WT124258.515.6467.68ΔSCO7613104211.734.7356.75ΔSCO7613+pHZ-7613113

	PHA(mg/L)	PHA (mg COD/L)	PHA (mmol C/L)	PHA (mg C/L)
WT	124	258.51	5.64	67.68
ΔSCO7613	104	211.73	4.73	56.75
ΔSCO7613+pHZ-7613	113	228.09	5.14	61.66

According to the FTIR analysis, pure PHB and PHB extracted from bacterial strains showed their strongest bands at 1721.2 cm^–1^ and near 1730 cm^–1^ corresponding to the ester carbonyl group, respectively (Figure 8). Although it was not possible to calculate the concentrations of PHB with the method we used, 1730 cm^–1^ band of mutant strain was seen much shorter compared to that of the wild type and the complemented strain. Other characteristic bands for PHB and PHB purified from bacterial strains were visible near 1279 cm^–1^ and 1178 cm^–1^ corresponding to the -CH group, respectively. These bands were totally disappeared in the mutant strain (Figure 8c).

### 3.4. Protein-protein interaction network analysis by STRING 

Since the mutation did not completely stop PHA production and no other putative phaC gene could be found in the genome, the effect of the mutation was thought to be indirect. By using STRING (Search Tool for the Retrieval of Interacting Genes/Proteins) database functional partners of SCO7613 product has been predicted (Figure 9). The protein that we are interested in has been found to interact with some regulatory proteins including Aba-like regulatory protein (SCO7614), TetR family transcriptional regulator (SCO0745), LuxR family regulator (SCO7295), etc. Aba-like regulatory protein (SCO7614) coding sequence is the neighbor of SCO7613 and it contains TTA leucine codon which is a possible target for bldA regulation. Other functional partners of SCO7613 product were predicted to be an essential cell division protein FtsQ (SCO2083) and a probable transcriptional regulator similar to *Streptomyces griseus* glycerol operon regulatory protein GylR (SCO7618). 

## 3. Discussion

*Streptomyces* are indicated as potential sources for the production of PHAs that can be used in biomedical applications and for the production of PHAs with desirable characteristics (Krishnan et al., 2017; Valappil et al., 2007; Williams et al., 1983). However very little is known about the biosynthesis and responsible biosynthetic genes in these organisms. Similarly, *S. coelicolor* A3(2) was announced to have a high potential to be transformed into an important PHA producer (Kalia et al., 2007), however *pha* C gene that encodes the key enzyme PHA synthase in the metabolic pathway is not determined in the genome of this organism. Likewise, although the presence of two main enzymes, PhaA (acetyl-CoA acetyltransferase or β-ketothiolase) and PhaB (acetoacetyl-CoA reductase), of the polyhydroxybutyrate pathway, have been shown before in *S. coelicolor* cell-free extracts, their genes were not determined in the genome (Packter and Flatman, 1983).

In this study, we aimed to identify *pha* C gene of *S. coelicolor.* BLAST searches of GeneBank database indicated that SCO7613 genomic region of *S. coelicolor* A3(2) shows similarity with the *pha* C synthase gene of *S. aureofaciens* . Deduced amino acid sequence of SCO7613 showed the presence of conserved lipase box-like sequence, ^555^GASAG^559^, in which serine residue was present as the conserved amino acid in the lipase box instead of cystein similar to *S. aureofaciens pha* C. To the best of our knowledge, there was no other region in the genome of *S. coelicolor* that shows strong similarities with the known *pha* C synthases. Rehm and Steinbüchel (1999) compared 30 PHA synthases from various bacteria and revealed that these enzymes exhibit similarity between 21% and 88%. SCO7613 and *pha* C of *S. aureofaciens* gave 44% similarity in their amino acid sequence so we choose SCO7613 as our target. First SCO7613 was deleted from *S. coelicolor* genome and the influence of this mutation on PHA biosynthesis was analysed by spectrophotometry, GC and FTIR. The deletion of this region from the genome decreased the PHA concentration in the cell, however, it did not cease the biosynthesis of PHA. Complementation of the mutation with pHZ-7613 plasmid showed that SCO7613 is responsible for the decrease in PHA biosynthesis. 

PHA amount determined by spectrophotometric method and GC analysis were not parallel to each other. This is most probably because of the difference in PHA purification methods. For spectrophotometric and FTIR analysis intracellular polymer is dissolved in organic solvents and high temperature increased the solubility of PHB. However, for GC analysis the cell pellets were directly freeze-dried and used for the PHA measurement which resulted in a low amount of PHA. Aramvash et al. (2018) have shown the efficiency of different organic solvents to dissolve intracellular PHAs andalsothe importance of temperature in the extraction of these polymers. Our results are supporting their findings.

According to spectrophotometric results, deletion of SCO7613 considerably decreased the PHA concentration, however it did not cease the biosynthesis of PHA. This can be explained by the presence of a second copy of *pha* C gene in the genome or the decrease in PHA content may be an indirect effect of the mutation. To the best of our knowledge, there was no other region similar to *pha* C gene in the genome of *S. coelicolor* . Moreover, it is known that PhaC synthase activity is based on the catalytic triad cysteine-histidine-aspartate. But, these conserved amino acids of PhaC were not present around the putative lipase box as part of the catalytic triad needed for synthase function. Therefore, we focused on the possibility that the decrease in the PHA concentration may be an indirect effect of the mutation. By using STRING database functional partners of SCO7613 product was predicted to be some important regulatory proteins like TetR family transcriptional regulator, LuxR family regulator, Aba-like regulator and a probable transcriptional regulator similar to *Streptomyces griseus* glycerol operon regulatory protein GylR. gylCABX operon is responsible for the glycerol catabolism in *S. coelicolor* and GylR is the negative autoregulator of this operon (Hindle and Smith, 1994). Possible interaction with GylR regulator made us ask the question that “Does the SCO7613 is a putative integral membrane protein responsible for glycerol transport?”. If this is the case then the decrease in PHA content can be explained by low precursor flow in the absence of SCO7613. Further research is needed to prove this hypothesis and to determine *pha* C gene of *S. coelicolor* which is declared as a potential PHA producer for biomedical applications.
